# Serum Alkaline Phosphatase and Risk of Incident Cardiovascular Disease: Interrelationship with High Sensitivity C-Reactive Protein

**DOI:** 10.1371/journal.pone.0132822

**Published:** 2015-07-13

**Authors:** Setor K. Kunutsor, Stephan J. L. Bakker, Jenny E. Kootstra-Ros, Ronald T. Gansevoort, John Gregson, Robin P. F. Dullaart

**Affiliations:** 1 Department of Public Health and Primary Care, University of Cambridge, Cambridge, United Kingdom; 2 School of Clinical Sciences, University of Bristol, Bristol, United Kingdom; 3 Department of Internal Medicine, University of Groningen and University Medical Center, Groningen, The Netherlands; 4 Top Institute Food and Nutrition, Wageningen, The Netherlands; 5 Department of Clinical Chemistry, University of Groningen and University Medical Center, Groningen, The Netherlands; 6 Department of Medical Statistics, London School of Hygiene and Tropical Medicine, London, United Kingdom; 7 Department of Endocrinology, University of Groningen and University Medical Center, Groningen, The Netherlands; Los Angeles, UNITED STATES

## Abstract

**Background:**

Alkaline phosphatase (ALP) has been suggested to be associated with cardiovascular disease (CVD) risk, however, important aspects of the association, such as shape and independence from established risk factors, have yet to be characterized in detail. We assessed the association of ALP with CVD risk and determined its utility for CVD risk prediction.

**Methods:**

Alkaline phosphatase activity was measured at baseline in the PREVEND prospective cohort involving 6,974 participants aged 28-75 years without pre-existing CVD. Hazard ratios (95% confidence intervals [CI]) and measures of risk discrimination and reclassification were assessed.

**Results:**

During a median follow-up of 10.5 years, 737 participants developed CVD. Serum ALP was correlated with several risk markers for CVD, with strongest correlations for age (r = 0.30; *P* < 0.001), gamma-glutamyltransferase (r = 0.26; *P* < 0.001), and C-reactive protein (CRP) (r = 0.25; *P* < 0.001). There was a non-linear “J-shaped” relationship between ALP and CVD risk. In analyses adjusted for conventional risk factors, the hazard ratio (95% CI) for CVD in a comparison of the top quintile versus bottom quintiles 1-4 of ALP values was 1.34 (1.14 to 1.56; *P*<0.001), which persisted after additional adjustment for potential confounders 1.33 (1.13 to 1.55; *P*<0.001). However, the association was somewhat attenuated after adjustment for CRP 1.24 (1.05 to 1.45; *P*=0.009). Addition of information on ALP to a CVD risk prediction model containing established risk factors did not improve the C-index or net reclassification.

**Conclusions:**

Available evidence suggests a non-linear association between ALP activity and CVD risk, which is partly dependent on CRP. Taking account of conventional risk factors, additional information on ALP does not improve CVD risk assessment.

## Introduction

Alkaline phosphatase (ALP) is a hydrolase enzyme, which is widely expressed in human tissues, but is highly concentrated in the liver, bone, and kidney [1]. Although its exact physiological function is unclear, serum ALP activity has commonly been used in clinical practice as a marker of hepatobiliary and bone disease [2]. Alkaline phosphatase is an inflammatory mediator like C-reactive protein (CRP) (a novel risk marker for cardiovascular disease [3]). Both ALP and CRP have consistently been shown to be directly and significantly associated with each other, with suggestions that they share common biological pathways [4–6]. Over the past decade, serum ALP has sparked interest as an emerging marker for cardiovascular risk in the general population, but uncertainty exists because important questions pertaining to its association with CVD remain unresolved. A limited number of population-based prospective cohort studies have generally suggested a positive association [2, 7–9], but such studies were often poorly powered or unable to adjust for potentially relevant confounders. For these reasons, the nature, magnitude, and independence of the association remain unclear. Furthermore, the majority of these studies were conducted in selected populations such as in the elderly or participants with coexisting morbidities [2, 7–9]. In our recently published literature-based meta-analysis of studies assessing the associations of liver enzymes and CVD risk in participants recruited from approximately general populations, the results suggested a modest positive linear association between ALP activity and CVD risk [10]. However, because only limited published studies with few data points were available for pooling, the uncertainties existing for ALP could not be adequately addressed.

With the emerging interest in the potential value of ALP activity in CVD risk prevention, there is a need to provide robust evidence on the association of baseline ALP values with risk of future CVD events in the general population using long-term observational data. Our primary objective was to characterize and quantify the nature and magnitude of the prospective association between ALP activity and risk of CVD using a large population-based sample of 6,974 participants free from CVD at entry. In a recent report, Wannamethee and colleagues reported considerable weakening of the association between ALP and CVD, following adjustment for CRP [7]. Therefore, to put the interdependence between ALP and CRP levels into clinical perspective, we also aimed to determine whether the ALP-CVD relationship is confounded or modified by CRP. Finally, we aimed to investigate for the first time, the extent to which ALP measurements could improve the prediction of first-onset CVD outcomes in general population settings when added to a conventional risk prediction model.

## Methods

### Study participants

For this study, we used primary data from the Prevention of Renal and Vascular End-stage Disease (PREVEND) study, an observational, general population based cohort study in the Netherlands, which began in 1997. Details of the study design and recruitment have been described in detail elsewhere [11]. Briefly, this prospective study was designed to investigate the natural course of urinary albumin excretion and its relation to renal and cardiovascular disease. The actual PREVEND cohort (N = 8,592) was recruited from inhabitants (aged 28–75 years) of the city of Groningen, the Netherlands. Baseline measurements were performed between 1997 and 1998. Baseline measurements were performed between 1997 and 1998. Participants with a history of prevalent CVD, liver disease, renal disease, or malignancy were excluded. The final cohort for this analysis included 6,974 subjects with non-missing information on ALP values and several CVD risk markers. The PREVEND study has been approved by the medical ethics committee of the University Medical Center Groningen and was conducted in accordance with the Declaration of Helsinki. Individual written informed consent was obtained from all participants, which was documented in a consent form approved by the medical ethics committee.

### Risk factor assessment

Participants underwent two outpatient visits to assess baseline data on demographics, anthropometric measurements, and cardiovascular risk factors. Venous blood was obtained after an overnight fast and 15 minutes of rest. Plasma samples were prepared by centrifugation at 4°C. Sera was stored at -20°C and heparinized plasma samples stored at -80°C until analysis. Plasma glucose was measured by dry chemistry (Eastman Kodak, Rochester, New York). Total cholesterol, high-density lipoprotein cholesterol (HDL-C), triglycerides, alanine aminotransferase (ALT), gamma-glutamyltransferase (GGT), and high sensitivity C-reactive protein (hsCRP) were measured as previously described [12, 13]. Serum creatinine was determined by Kodak Ektachem dry chemistry (Eastman Kodak, Rochester, New York) and serum cystatin C level by nephelometry (BN II N) (Dade Behring Diagnostic, Marburg, Germany). In a validation experiment, we showed that serum creatinine levels as measured by dry chemistry are comparable to levels measured by enzymatic method. Urinary albumin excretion (UAE) was calculated as the mean of two 24-hour urine collections. Estimated glomerular filtration rate (eGFR), was calculated using the Chronic Kidney Disease Epidemiology Collaboration (CKD-EPI) combined creatinine-cystatin C equation [14]. Serum ALP activity was measured using a standardized enzymatic method on a Roche Modular P analyzer (Roche, Mannheim, Germany) according to the recommendations of the International Federation of Clinical Chemistry [15]. The reference ranges for ALP values employed by our laboratory was < 98 U/L for females and < 115 U/L for males. Hypertension was defined as systolic blood pressure (SBP) of ≥140 mm Hg, a diastolic blood pressure (DBP) of ≥90 mm Hg, or use of antihypertensive medication according to self-report or to pharmacy data. Diabetes was defined as a fasting glucose level of ≥ 7.0 mmol/l, a nonfasting glucose level of ≥11.1 mmol/l or use of antidiabetic medication according to self-report or to pharmacy data [16].

### Endpoint ascertainment

The primary outcome was first-onset CVD events (morbidity and mortality) with subsidiary analyses of incident coronary heart disease (CHD) and stroke outcomes. Date and cause of death were obtained by record linkage with the Dutch Central Bureau of Statistics, while information on hospitalization for cardiovascular morbidity was received from PRISMANT, the Dutch national registry of hospital discharge diagnoses. The validity of this database has been shown to be good, with 84% of primary diagnoses and 87% of secondary diagnoses matching the diagnoses recorded in patients’ charts [17, 18]. All data were coded according to the *International Classification of Diseases*, Ninth Revision (ICD-9) and the classification of interventions. Cardiovascular outcomes were defined as the combined incidence of acute myocardial infarction (ICD-9 code 410), acute and subacute ischemic heart disease (ICD-9 code 411), coronary artery bypass grafting (ICD-9 code 414) or percutaneous transluminal coronary angioplasty, subarachnoid hemorrhage (ICD-9 code 430), intracerebral hemorrhage (ICD-9 code 431), other intracranial hemorrhage (ICD-9 code 432), occlusion or stenosis of the precerebral (ICD-9 code 433) or cerebral (ICD-9 code 434) arteries, and other vascular interventions such as percutaneous transluminal angioplasty or bypass grafting of peripheral vessels (ICD-9 code 440) and aorta (ICD-9 code 441).

### Statistical analyses

The principal analyses were pre-specified to exclude participants with a history of CVD at baseline. The primary outcome was first-onset CVD, defined as fatal or nonfatal CHD event or any stroke. Positively skewed variables (e.g., ALT, ALP, GGT, triglycerides, hsCRP, creatinine, and UAE) were natural log-transformed to achieve approximately normal distributions. We performed descriptive analyses summarizing baseline characteristics of participants. Cross-sectional associations of ALP with risk markers for CVD were assessed using linear regression models adjusted for age and sex. Time-to-event analyses were conducted using Cox proportional hazards models [19] to examine the association of baseline ALP values with risk of CVD after confirming assumptions of proportionality of hazards [20]. The shape of the association of ALP values with CVD risk was assessed by plotting hazard ratios (HRs) calculated within quintiles of baseline ALP values against the mean log_e_ ALP value within each quintile using floating absolute risks (FARs) [21]. As the association showed a non-linear shape, ALP was not modelled continuously, but entered as fifths defined according to its baseline distribution. Because of the relatively flat risk of CVD across quintiles 1–4, these categories were combined and served as the reference comparison. Hazard ratios were adjusted for established CVD risk factors [age, sex, smoking status, history of diabetes, SBP, total cholesterol, and HDL-C] and further for body mass index (BMI), alcohol consumption, fasting glucose, triglycerides, eGFR, UAE, and hsCRP. We performed subgroup analyses using interaction tests to assess statistical evidence of any differences in HRs across categories of pre-specified individual characteristics (such as age, sex, history of diabetes, smoking status, alcohol consumption, BMI, SBP, total cholesterol, HDL-C, triglycerides, eGFR, UAE, and hsCRP). Subsidiary analyses included further adjustment for other liver enzymes, GGT and ALT. To avoid potential bias due to reverse causation, we carried out subsidiary analyses that excluded participants with a diagnosis of diabetes at baseline, CVD events ascertained in the first two years of follow-up, participants on regular anti-hypertensive medication, and participants on regular lipid-lowering medication (statins). Subsidiary analysis also involved excluding participants with increased albuminuria (UAE ≥ 30 mg/24 hours), as it is a well-established cardiovascular risk marker [22]. Finally, we conducted sensitivity analyses which employed the use of complex survey design analyses [23], taking into account that the PREVEND cohort is oversampled for subjects with higher albuminuria levels, thereby enabling the results to be extrapolated to the broader (general) population.

To assess whether adding information on ALP values to conventional cardiovascular risk factors is associated with improvement in prediction of CVD risk, we calculated measures of discrimination and reclassification [24, 25] for censored time-to-event data (Harrell’s C-index [26]). Since recently developed risk prediction scores [27, 28] predict a composite CVD endpoint (combining outcomes of CHD and stroke) and because these outcomes share common risk factors and treatments, the primary outcome for our risk prediction analysis was any first CVD event. To investigate the change in C-index on addition of ALP, we added ALP to a model based on risk factors included in the Framingham CVD Risk Score (i.e., age, sex, smoking status, SBP, total cholesterol, and HDL-C) [29]. For participants with at least 10 years of follow-up, reclassification was assessed using the net-reclassification-improvement (NRI) [24, 25], by comparing the cardiovascular risk from the model containing conventional risk factors to the predicted risk from the model containing conventional risk factors plus ALP. Reclassification analysis was based on predicted 10-year CVD risk categories of low (<5%), intermediate (5 to <7.5%), and high (≥7.5%) risk [30]. Risk discrimination and reclassification analyses based on the Reynolds Risk Score (RRS) [31, 32], which has been shown to be better calibrated for major CVD events than the Framingham CVD model [33] were also performed in subsidiary analyses. Risk prediction analysis was restricted to participants without a known history of diabetes at baseline. All statistical analyses were conducted using Stata version 13 (Stata Corp, College Station, Texas) and P values were 2-sided.

## Results

### Baseline characteristics and correlates of ALP

Overall, the mean age at baseline of the 6,974 participants eligible for the present study was 48 (SD 12) years and 52% were women. Median (interquartile range) ALP value was 62 (24) U/L. Baseline descriptive characteristics of the participants are shown in Table 1. Log_e_ ALP values were modestly and positively correlated with age (r = 0.30), log_e_ GGT (r = 0.26), and log_e_ hsCRP (r = 0.25). There were weak and positive correlations with physical measures (BMI, waist circumference, and blood pressure), as well as several lipid, metabolic, and renal function (cystatin C and UAE) markers. Inverse correlations were observed for HDL-C and log_e_ creatinine. Baseline ALP values were higher by 8% in men compared with women, by 9% in people with diabetes compared with non-diabetics, and lower by 7% in current alcohol drinkers compared with non-current alcohol drinkers (Table 1). Clinical and laboratory data of participants by ALP quintiles are shown in S1 Table.

**Table 1:** Baseline participant characteristics and cross-sectional correlates of alkaline phosphatase.

	Mean (SD) or %	Pearson correlation r (95% CI)†	Percentage difference (95% CI) in ALP levels per 1 SD higher or compared to reference category of correlate‡
Log_e_ ALP (U/L)	4.12 (0.29)	-	-
***Questionnaire***			
Sex			
Female	51.7		Ref
Male	48.3		8% (7, 10)***
Age at survey (years)	48 (12)	0.30 (0.28, 0.32)***	9% (8, 10)***
History of diabetes			
No	97.0	-	Ref
Yes	3.0	-	9% (5, 13)***
Smoking status			
Non-smokers	30.6	-	Ref
Current smokers	69.4	-	0% (-1, 2)
Alcohol consumption			
Non-consumers	24.4	-	Ref
Current consumers	75.6	-	-7% (-8, -6)***
History of hypertension			
No	90.1	-	Ref
Yes	9.9	-	-1% (-3, 1)
Regular use of anti-hypertensive medication			
No	89.1	-	Ref
Yes	10.9	-	0% (-2, 2)
Regular use of diabetic medication			
No	99.0	-	Ref
Yes	1.0	-	0% (-6, 7)
Regular use of lipid-lowering medication			
No	97.5	-	Ref
Yes	2.5	-	3% (-1, 8)
***Physical measurements***			
BMI (kg/m^2^)	26.0 (4.2)	0.15 (0.13, 0.17)***	4% (4, 5)***
Waist circumference (cm)	87.8 (13.0)	0.17 (0.14, 0.19)***	5% (5, 6)***
SBP (mmHg)	128.2 (19.9)	0.14 (0.12, 0.17)***	5% (4, 5)***
DBP (mmHg)	73.7 (9.7)	0.11 (0.09, 0.13)***	3% (3, 4)***
***Lipid markers***			
Total cholesterol (mmol/l)	5.63 (1.12)	0.12 (0.10, 0.14)***	4% (3, 4)***
HDL-C (mmol/l)	1.34 (0.40)	-0.17 (-0.19, -0.15)***	-5% (-6, -4)***
Log_e_ triglycerides (mmol/l)	0.18 (0.53)	0.17 (0.14, 0.19)***	5% (4, 6)***
Apo AI (g/l)	1.39 (0.30)	-0.11 (-0.14, -0.09)***	-4% (-4, -3)***
Apo B (g/l)	1.03 (0.31)	0.12 (0.09, 0.14)***	3% (3, 4)***
***Metabolic*, *inflammatory*, *and renal function markers***			
Log_e_ hsCRP (mg/l)	0.23 (1.17)	0.25 (0.22, 0.27)***	7% (6, 8)***
Fasting plasma glucose (mmol/l)	4.82 (1.11)	0.11 (0.08, 0.13)***	3% (2, 4)***
Log_e_ creatinine (μmol/1)	4.41 (0.17)	-0.06 (-0.09, -0.04)***	-2% (-3, -1)***
Cystatin C (mg/l)	0.79 (0.19)	0.08 (0.06, 0.10)***	2% (2, 3)***
eGFR (ml/min/1.73 m^2^)	100.9 (39.7)	-0.01 (-0.03, 0.01)***	-0% (-1, 0)
Log_e_ UAE (mg/24 hours)	2.47 (0.96)	0.04 (0.01, 0.06)***	1% (0, 2)*
Log_e_ GGT (U/L)	3.21 (0.63)	0.26 (0.23, 0.28)***	8% (7, 9)***
Log_e_ ALT (U/L)	3.03 (0.48)	0.19 (0.17, 0.22)***	6% (5, 7)***

ALT, alanine aminotransferase; Apo AI, apolipoprotein AI; Apo B, apolipoprotein; BMI, body mass index; hsCRP, high sensitivity C-reactive protein; DBP, diastolic blood pressure; eGFR, estimated glomerular filtration rate (as calculated using the Chronic Kidney Disease Epidemiology Collaboration combined creatinine-cystatin C equation); ALP, alkaline phosphatase; GGT, gamma-glutamyltransferase; HDL-C, high-density lipoprotein cholesterol; Ref, reference; SD, standard deviation; SBP, systolic blood pressure; UAE, urinary albumin excretion

^†^Pearson correlation coefficients between log_e_ ALP and the row variables;

^‡^Percentage change in ALP values per 1 SD increase in the row variable (or for categorical variables, the percentage difference in mean ALP values for the category versus the reference) adjusted for age and sex; asterisks indicate the level of statistical significance:

*, p<0.05;

**, p<0.01;

***, p<0.001

### ALP levels and risk of incident CVD

**Fig 1:**
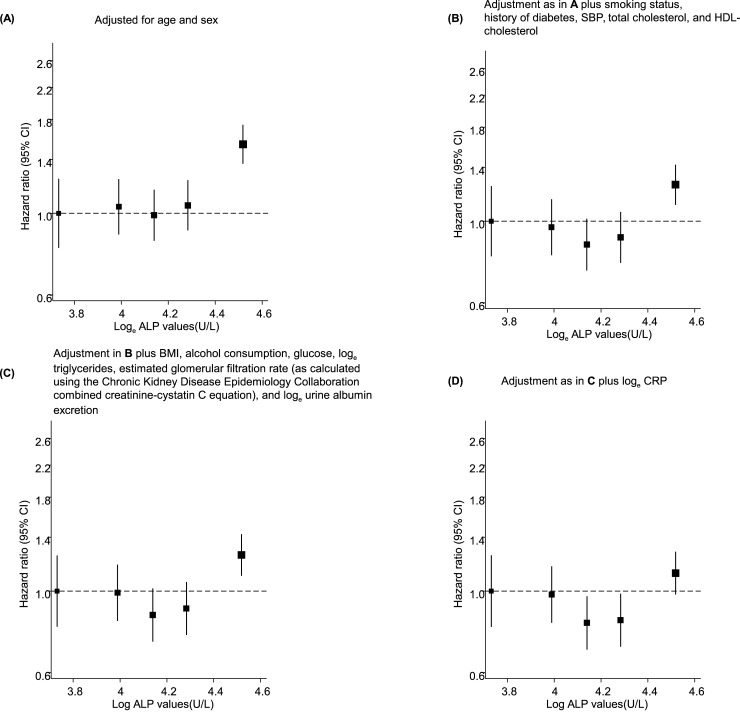
Hazard ratios for incident cardiovascular disease by baseline values of log_e_ alkaline phosphatase using floating absolute risks. **A**, adjusted for age and sex; **B**, adjustment as in A plus smoking status, history of diabetes, systolic blood pressure, total cholesterol, and high-density lipoprotein cholesterol; **C**, adjustment as in B plus body mass index, alcohol consumption, glucose, log_e_ triglycerides, estimated glomerular filtration rate (as calculated using the Chronic Kidney Disease Epidemiology Collaboration combined creatinine-cystatin C equation), and log_e_ urine albumin excretion; **D**, adjustment as in C plus log_e_ C-reactive protein; the size of the box is proportional to the inverse of the variance of hazard ratio.

**Fig 2:**
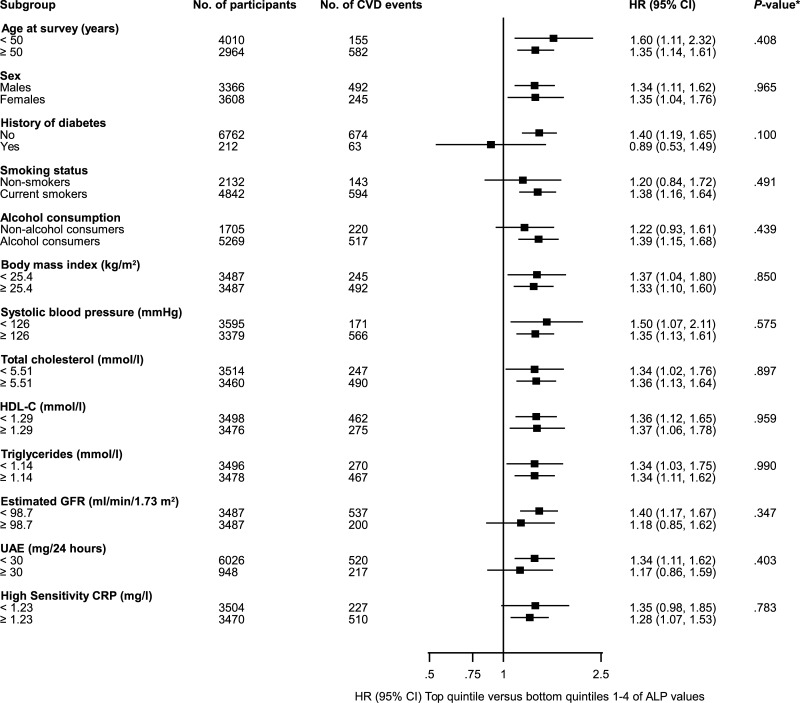
Hazard ratios for cardiovascular disease comparing top quintile versus bottom quintiles 1–4 of baseline ALP values, by several participant level characteristics. Hazard ratios were adjusted for age, sex, smoking status, history of diabetes, systolic blood pressure, total cholesterol, and high-density lipoprotein cholesterol (HDL-C); CI, confidence interval (bars); CRP, C-reactive protein; CVD, cardiovascular disease; Estimated GFR, glomerular filtration rate (as calculated using the Chronic Kidney Disease Epidemiology Collaboration combined creatinine-cystatin C equation); HR, hazard ratio; UAE, urinary albumin excretion; *, *P*-value for interaction; Cut-offs used for body mass index, total cholesterol, HDL-C, triglycerides, estimated GFR, and high sensitivity CRP are median values.

During 65,041 person years at risk (median follow-up of 10.5 years), 737 incident CVD events (annual rate 11.2/1000 person-years at risk) were recorded. In analyses adjusted for age and sex, a non-linear approximately “J-shaped” relationship was observed between ALP values and CVD risk. The shape of the association was similar on adjusting for several established risk factors (smoking status, history of diabetes, SBP, total cholesterol, and HDL-C,) and further for BMI, alcohol consumption, glucose, log_e_ triglycerides, eGFR, log_e_ UAE, and log_e_ hsCRP (Fig 1). Further analyses compared the top quintile versus bottom quintiles 1–4 of the ALP distribution. The age- and sex-adjusted HR for CVD was 1.51 (95% CI: 1.29 to 1.76; *P*<0.001), which changed to 1.34 (95% CI: 1.14 to 1.56; *P*<0.001) following further adjustment for conventional CVD risk factors. The results remained materially unchanged after additional adjustment for BMI, alcohol consumption, glucose, log_e_ triglycerides, eGFR, and log_e_ UAE 1.33 (95% CI: 1.13 to 1.55; *P*<0.001). However, further adjustment for log_e_ hsCRP somewhat attenuated the association 1.24 (95% CI: 1.05 to 1.45; *P* = 0.009) (Table 2). HRs did not change importantly in analyses that excluded CVD outcomes recorded in the first two years of follow-up, participants with a history of diabetes at baseline, participants on regular anti-hypertensive medication, participants on regular lipid-lowering medication, or participants with UAE ≥ 30 mg/24 hours (S2 Table). Also, the associations generally did not vary significantly by levels or categories of several clinically relevant characteristics and other risk markers (*P* for interaction ≥ 0.10 for each; Fig 2). In an age- and sex-adjusted analysis, the ALP-CVD association was somewhat attenuated after single additional adjustment for log_e_ hsCRP (S3 Table). There was no evidence of statistically significant effect modification by hsCRP on the association in age- and sex-adjusted analysis (*P* for interaction = 0.58). In separate analyses for CHD and stroke, the initial positive association of ALP activity with CHD in analyses adjusted for several established risk factors and potential confounders lost significance upon further adjustment for hsCRP. With respect to the shape of the ALP-CHD relationship, there was a continuous association in analysis adjusted for age and sex, potentially consistent with a curvilinear shape, which was altered (almost flat) on further adjustment for other covariates (S1 Fig). Further work is required to determine the shape that better describes the association. There was no significant evidence of an association with stroke in all models (Table 2). All the results remained similar on further adjustment for GGT and ALT (S4 Table). To put the strength of the associations of ALP levels with CVD, CHD, and stroke risk into context, comparisons were made to the associations of hsCRP with these same outcomes. As expected, there were significant associations of hsCRP with all cardiovascular outcomes and were of comparable strength (Table 3). All results were essentially similar when design-based Cox regression analyses were performed (data not shown).

**Table 2:** Association of alkaline phosphatase with incident cardiovascular disease, coronary heart disease, and stroke.

Quintiles of ALP	Events / Total	Model 1		Model 2		Model 3		Model 4	
		HR (95% CI)	*P-*value	HR (95% CI)	*P-*value	HR (95% CI)	*P-*value	HR (95% CI)	*P-*value
Cardiovascular disease									
Q1 –Q4	486 / 5,601	ref		ref		ref		ref	
Q5	251 / 1,373	1.51 (1.29 to 1.76)	< 0.001	1.34 (1.14 to 1.56)	< 0.001	1.33 (1.13 to 1.55)	< 0.001	1.24 (1.05 to 1.45)	0.009
Coronary heart disease									
Q1 –Q4	262 / 5,601	ref		ref		ref		ref	
Q5	136 / 1,373	1.53 (1.24 to 1.89)	< 0.001	1.29 (1.05 to 1.60)	0.017	1.30 (1.05 to 1.61)	0.016	1.22 (0.98 to 1.52)	0.075
Stroke									
Q1 –Q4	108 / 5,601	ref		ref		ref		ref	
Q5	48 / 1,373	1.19 (0.84 to 1.68)	0.319	1.06 (0.75 to1.50)	0.748	1.03 (0.73 to 1.47)	0.862	0.92 (0.65 to 1.32)	0.654

ALP,alkaline phosphatase; Q, quintile

Model 1: Age and sex

Model 2: Model 1 plus smoking status, history of diabetes, systolic blood pressure, total cholesterol, and high-density lipoprotein-cholesterol

Model 3: Model 2 plus body mass index, alcohol consumption, glucose, log_e_ triglycerides, estimated glomerular filtration rate (as calculated using the Chronic Kidney Disease Epidemiology Collaboration combined creatinine-cystatin C equation), and log_e_ urinary albumin excretion

Model 4: Model 3 plus log_e_ C-reactive protein.

**Table 3:** Associations of hsCRP with incident CVD, CHD and Stroke.

Models	CVD		CHD		Stroke	
	Hazard ratio (95% CI)	*P*-value	Hazard ratio (95% CI)	*P*-value	Hazard ratio (95% CI)	*P*-value
	6,974 participants and 737 cases		6,974 participants and 398 cases		6,974 participants and 156 cases	
Model 1	1.41 (1.30 to 1.52)	< 0.001	1.43 (1.30 to 1.59)	< 0.001	1.46 (1.23 to 1.73)	< 0.001
Model 2	1.27 (1.17 to 1.38)	< 0.001	1.25 (1.11 to 1.41)	< 0.001	1.37 (1.14 to 1.64)	0.001
Model 3	1.26 (1.17 to 1.38)	< 0.001	1.24 (1.09 to 1.40)	0.001	1.41 (1.17 to 1.70)	< 0.001

Hazard ratios are reported per 1 standard deviation increase in log_e_ hsCRP levels; 1 standard deviation higher log_e_ hsCRP was approximately equivalent to three-fold higher hsCRP levels.

CHD, coronary heart disease; CVD, cardiovascular disease; hsCRP, high sensitivity C-reactive protein

Model 1: Age and sex

Model 2: Model 1 plus smoking status, history of diabetes, systolic blood pressure, total cholesterol, and high-density lipoprotein-cholesterol

Model 3: Model 2 plus body mass index, alcohol consumption, glucose, log_e_ triglycerides, estimated glomerular filtration rate (as calculated using the Chronic Kidney Disease Epidemiology Collaboration combined creatinine-cystatin C equation), and log_e_ urinary albumin excretion.

### ALP and CVD risk prediction

A CVD risk prediction model containing established risk factors yielded a C-index of 0.7843 (95% CI: 0.7689 to 0.7996). After addition of information on ALP values, the C-index was 0.7846 (95% CI: 0.7692 to 0.8000), representing a marginal increase of 0.0003 (95% CI: -0.0015 to 0.0022; *P* = 0.72) (Table 4). There were no significant differences in cardiovascular risk discrimination according to individual level clinically relevant characteristics (S2 Fig). In addition, there was no significant improvement in the classification of participants into predicted 10-year CVD risk categories (NRI: 0.20%, -1.19 to 1.58%; *P* = 0.78). There was no significant improvement in risk discrimination and reclassification when the model containing the RRS components was used: C-index change of 0.0001 (95% CI: -0.0010 to 0.0011; *P* = 0.96) and NRI of -0.35% (-1.60 to 0.89%; *P* = 0.58) (S5 Table).

**Table 4:** Risk discrimination and reclassification upon addition of ALP to the Framingham CVD risk prediction model containing conventional risk factors.

**Discrimination**	
C-index (95% CI): conventional risk factors	0.7843 (0.7689 to 0.7996)
C-index (95% CI): conventional risk factors plus ALP	0.7846 (0.7692 to 0.8000)
C-index change (95% CI)	0.0003 (-0.0015 to 0.0022)
*P*-value	0.72
**Reclassification**	
*Participants who did not develop CVD at 10 years*	
Appropriately reclassified	112 (2.39%)
Inappropriately reclassified	110 (2.35%)
No change	4,465 (95.26%)
*Participants who developed CVD at 10 years*	
Appropriately reclassified	9 (1.37%)
Inappropriately reclassified	8 (1.22%)
No change	639 (97.41%)
Net reclassification index (95% CI)	0.20% (-1.19% to 1.58%)
*P*-value	0.78

The model with conventional risk factors included age, sex, smoking status, systolic blood pressure, total cholesterol, and high-density lipoprotein cholesterol; CVD, cardiovascular disease; ALP, alkaline phosphatase.

## Discussion

In this large-scale population-based study of individuals without a history of CVD at baseline, there were generally modest positive associations of ALP values with several cardiovascular risk markers. We observed a non-linear approximately “J-shaped” association between ALP and CVD risk in analyses adjusted for a comprehensive panel of established and potential confounders. Our finding of a non-linear relationship between ALP and CVD risk is compatible with some previous reports. In the 20th year follow-up examination and analyses of the British Regional Heart Study (BRHS), the results were suggestive of a non-linear association between ALP and stroke/CVD events in older men aged 60 to 79 years [7]. In their 16 years follow-up of over 10,000 participants aged 40–69 in the Circulatory Risk in Communities Study (CIRCS), Shimizu and colleagues demonstrated a “U-shaped” association between ALP values and stroke risk in both men and women [34]. Our data also suggest that baseline circulating ALP is positively associated with first-ever CVD outcomes, independent of several established risk factors plus potential confounders including other liver enzymes (GGT and ALT); however, the association was somewhat attenuated on further adjustment for hsCRP. The results remained generally consistent across several clinically relevant subgroups and at different levels of risk factors including hsCRP. The associations were also similar in several sensitivity analyses. A significant association with CHD was observed in analyses adjusted for several established risk factors and potential confounders but was attenuated after further adjustment for hsCRP, consistent with findings from the BRHS. There was however no significant evidence of an association with stroke, which was also consistent with the BRHS [7], but in contrast to that observed in CIRCS [34]. The differences in findings may be attributed to differences in statistical power. In CIRCS [34], there was almost double the number of stroke cases compared to our study or that of BRHS, therefore there is a likelihood that the null results could be due to the low stroke event rates. In addition, given the different aetiologies for the endpoints of stroke and CVD in general [35], large-scale studies for the particular cardiovascular outcome of stroke are still warranted. In addition, our analyses indicate that addition of information on ALP values to conventional cardiovascular risk factors does not importantly improve CVD risk prediction.

### Possible explanations for findings

Potential mechanisms for increased cardiovascular risk in people with elevated values of ALP have been postulated. Alkaline phosphatase catalyses the hydrolysis of inorganic pyrophosphate, an inhibitor of vascular calcification [1], which leads to vascular hardening and promotes the atherosclerotic process [36]. In advanced atherosclerotic lesions, there is calcification with increased expression of ALP [37]. Mechanisms related to impaired vascular homeostasis or subclinical liver dysfunction have also been implicated [2, 34]. There is also a possibility that this could be due to underlying vitamin D or parathyroid hormone status. Low levels of vitamin D are usually associated with elevated ALP levels. Interestingly, a recent elegant study by Durup and colleagues has demonstrated a reverse J-shaped association between vitamin D (as measured by 25-hydroxyvitamin D) and CVD mortality [38]. We did not have data on vitamin D levels and therefore could not investigate this possibility. Additionally, ALP values are known to be elevated in obese patients and may play a role in adipogenesis [5, 39]. There are also suggestions that inflammation may be the common link between ALP and CVD development [2, 9]. Alkaline phosphatase is an acute phase reactant [40], which is associated with CRP [4, 5, 41], the most commonly used marker of low-grade chronic inflammation. Elevated values of ALP may therefore reflect inflammation of hepatic origin, given that CRP is secreted by the liver [7]. Our observed ALP-CVD association was materially attenuated after adjusting for hsCRP in both analyses initially only adjusted for age and sex and further for several conventional risk factors, which supports the hypothesis that inflammatory processes may play a role in the aetiology between ALP and CVD risk. In line with the recent report by Wannamethee and colleagues, the association between ALP and CVD was considerably weakened after adjustment for hsCRP [7]. If hsCRP mediates the association between ALP activity and risk of CVD, then correction for hsCRP could be an overadjustment. Evidence supportive of potential causal pathways may need to be confirmed in appropriate interventional studies or Mendelian randomization studies [42].

### Implications of findings

Our findings are relevant and may have clinical implications. They underscore a potentially deleterious role of increasing ALP activity on future risk of CVD/CHD in general populations. Elevations in ALP activity suggest decreased inhibition of vascular calcification, which is associated with myocardial infarction and coronary death [43] and is a significant risk factor in the pathogenesis of CVD. Results from a study in rat models have found evidence to support a substantial vascular calcification reduction effect of three novel inhibitors (5361418, 5923412, and 5804079) of the physiological pyrophosphate activity of ALP [44]. Therapies such as activated vitamin D products and calcimimetics have also been shown to lower levels of circulating ALP [45, 46]. Given that mitigation of vascular calcification is an emerging target in the treatment of atherosclerosis [9], further studies on ALP inhibitors and interventions that lower elevated activity of ALP are warranted. In the absence of such studies, individuals with increased ALP values may need further clinical evaluation and close monitoring.

### Strengths and limitations

The notable strengths include a large sample that was representative of the general population and had a long follow-up duration. There was information on a comprehensive panel of lifestyle and biological markers to allow adequate adjustment for potential confounding, enabling reliable assessments of the ALP-CVD association. In order to limit any possibilities of reverse-causation bias, this current study was designed to involve individuals free of prior vascular or malignant disorders at baseline. Our results remained robust in several sensitivity analyses. The reliability of the data was also confirmed by our ability to replicate the independent association of hsCRP with cardiovascular outcomes [3]. In addition to the several strengths enumerated, our study did have some limitations. It was not possible to correct the estimates for within-individual variation in values of ALP over time which can cause underestimation of associations, because repeat ALP measurements were not available. There is evidence to suggest that ALP values in individuals can fluctuate over time [47], hence, the associations demonstrated may be even stronger. Owing to the observational nature of data available, potential residual confounding due to errors in risk marker measurements and other unmeasured confounders (such as serum calcium, parathyroid hormone, and vitamin D) cannot be entirely ruled out. Assays for total circulating ALP were used, therefore it was not possible to evaluate which ALP isoenzyme [48] was associated with CVD risk.

## Conclusions

In conclusion, our data suggest a non-linear positive association between circulating ALP activity and CVD risk, which is partly dependent on CRP. Given knowledge of conventional risk factors, additional information on ALP does not improve CVD risk assessment.

## Supporting Information

S1 Fig Hazard ratios for incident coronary heart disease by baseline values of log_e_ alkaline phosphatase using floating absolute risks. (DOCX)

S2 Fig Change in Harrel’s C-index upon adding ALP values to conventional risk factors, by individual level characteristics. (DOCX)

S1 Table Baseline characteristics of the PREVEND cohort by ALP quintiles. (DOCX)

S2 Table Hazard Ratios for cardiovascular disease with first two years of follow-up, participants with history of diabetes, participants on regular antihypertensive medication, participants on regular lipid-lowering medication, and participants with UAE ≥ 30 mg/24 hours excluded. (DOCX)

S3 Table Age and sex-adjusted hazard ratios of ALP for incident cardiovascular diseases with additional adjustment for C-reactive protein. (DOCX)

S4 Table Association of alkaline phosphatase with incident cardiovascular disease, coronary heart disease, and stroke with further adjustment for other liver enzymes. (DOCX)

S5 Table Risk discrimination and reclassification upon addition of ALP to a CVD risk prediction model containing Reynolds Risk Score components. (DOCX)
