# Supplementary material for: Serum Alkaline Phosphatase and Risk of Incident Cardiovascular Disease: Interrelationship with High Sensitivity C-Reactive Protein
Source: PLoS One. 2015 Jul 13;10(7):e0132822. doi: 10.1371/journal.pone.0132822 (PMC4500413; doi:10.1371/journal.pone.0132822)
Supplement: S1 Fig — (DOCX) [file pone.0132822.s001.docx]

**S1 Fig.** **Hazard ratios for incident coronary heart disease by baseline values of log_e_ alkaline phosphatase using floating absolute risks**

**A**, adjusted for age and sex; **B**, adjustment as in A plus smoking status, history of diabetes, systolic blood pressure, total cholesterol, and high-density lipoprotein cholesterol; **C**, adjustment as in B plus body mass index, alcohol consumption, glucose, log_e_ triglycerides, estimated glomerular filtration rate (as calculated using the Chronic Kidney Disease Epidemiology Collaboration combined creatinine-cystatin C equation), and log_e_ urinary albumin excretion; **D**, adjustment as in C plus log_e_ C-reactive protein; the size of the box is proportional to the inverse of the variance of hazard ratio
