# Supplementary material for: Serum Alkaline Phosphatase and Risk of Incident Cardiovascular Disease: Interrelationship with High Sensitivity C-Reactive Protein
Source: PLoS One. 2015 Jul 13;10(7):e0132822. doi: 10.1371/journal.pone.0132822 (PMC4500413; doi:10.1371/journal.pone.0132822)
Supplement: S2 Fig — (DOCX) [file pone.0132822.s002.docx]

**S2 Fig. Change in Harrel’s C-index upon adding ALP values to conventional risk factors, by individual level characteristics**

The model with conventional risk factors included age, sex, smoking status, systolic blood pressure, total cholesterol, and high-density lipoprotein cholesterol; ALP, alkaline phosphatase; HDL-C, high-density lipoprotein cholesterol; CRP, C-reactive protein; GFR, glomerular filtration rate; UAE, urinary albumin excretion
