# Supplementary material for: Serum Alkaline Phosphatase and Risk of Incident Cardiovascular Disease: Interrelationship with High Sensitivity C-Reactive Protein
Source: PLoS One. 2015 Jul 13;10(7):e0132822. doi: 10.1371/journal.pone.0132822 (PMC4500413; doi:10.1371/journal.pone.0132822)
Supplement: S1 Table — (DOCX) [file pone.0132822.s003.docx]

**S1 Table.** **Baseline characteristics of the PREVEND cohort by ALP quintiles**

|  | **Quintile 1**  **(≤49 U/L)**  **Mean (SD), median (IQR), or %** | **Quintile 2**  **(50-58 U/L)**  **Mean (SD), median (IQR), or %** | **Quintile 3**  **(59-66 U/L)**  **Mean (SD), median (IQR), or %** | **Quintile 4**  **(67-78 U/L)**  **Mean (SD), median (IQR), or %** | **Quintile 5**  **(≥79 U/L)**  **Mean (SD), median (IQR), or %** |
| --- | --- | --- | --- | --- | --- |
| ***Questionnaire*** |  |  |  |  |  |
|  |  |  |  |  |  |
| Female | 68.7 | 53.2 | 45.2 | 43.1 | 46.9 |
| Male | 31.3 | 46.8 | 54.8 | 56.9 | 53.1 |
| Age at survey (years) | 42.8 (10.1) | 46.2 (11.7) | 48.8 (12.5) | 50.7 (12.6) | 53.7 (12.3) |
| History of diabetes | 1.1 | 2.0 | 2.7 | 3.2 | 6.3 |
| Ever smokers | 66.6 | 69.2 | 67.5 | 71.5 | 72.3 |
| Current alcohol consumers | 81.2 | 78.3 | 74.3 | 74.6 | 68.7 |
| History of hypertension | 6.4 | 8.4 | 10.2 | 11.5 | 13.5 |
| Regular use of anti-hypertensive medication | 6.5 | 9.2 | 11.1 | 13.4 | 14.8 |
| Regular use of diabetic medication | 0.5 | 0.8 | 1.3 | 0.9 | 1.6 |
| Regular use of lipid-lowering medication | 1.6 | 1.4 | 2.6 | 3.3 | 3.8 |
|  |  |  |  |  |  |
| ***Physical measurements*** |  |  |  |  |  |
| BMI (kg/m^2^) | 24.4 (3.7) | 25.4 (4.0) | 26.3 (4.2) | 26.7 (4.3) | 27.2 (4.4) |
| Waist circumference (cm) | 81.4 (12.0) | 85.7 (12.5) | 89.2 (12.9) | 90.9 (12.3) | 92.6 (12.0) |
| SBP (mmHg) | 119.5 (16.4) | 125.4 (18.1) | 129.0 (19.1) | 131.5 (19.8) | 136.4 (21.4) |
| DBP (mmHg) | 69.8 (8.9) | 72.8 (9.4) | 73.9 (9.0) | 75.2 (9.8) | 77.0 (9.8) |
|  |  |  |  |  |  |
| ***Lipid markers*** |  |  |  |  |  |
| Total cholesterol (mmol/l) | 5.27 (1.03) | 5.50 (1.07) | 5.66 (1.10) | 5.78 (1.12) | 5.96 (1.18) |
| HDL-C (mmol/l) | 1.48 (0.41) | 1.40 (0.42) | 1.29 (0.37) | 1.27 (0.38) | 1.22 (0.37) |
| Triglycerides (mmol/l)* | 0.94 (0.57) | 1.05 (0.69) | 1.16 (0.82) | 1.25 (0.97) | 1.38 (1.00) |
| Apo AI (g/l) | 1.46 (0.30) | 1.41 (0.32) | 1.38 (0.28) | 1.35 (0.29) | 1.35 (0.28) |
| Apo B (g/l) | 0.93 (0.29) | 0.98 (0.29) | 1.04 (0.30) | 1.07 (0.32) | 1.11 (0.32) |
|  |  |  |  |  |  |
| ***Metabolic, inflammatory, and renal function markers*** |  |  |  |  |  |
| hsCRP (mg/l)* | 0.77 (1.39) | 0.88 (1.65) | 1.31 (2.14) | 1.45 (2.61) | 2.15 (3.79) |
| Fasting plasma glucose (mmol/l) | 4.53 (0.72) | 4.70 (0.91) | 4.80 (0.89) | 4.91 (1.17) | 5.17 (1.56) |
| Creatinine (µmol/1)* | 0.89 (0.18) | 0.92 (0.20) | 0.93 (0.19) | 0.95 (0.20) | 0.94 (0.23) |
| Cystatin C (mg/l) | 0.74 (0.19) | 0.77 (0.19) | 0.79 (0.19) | 0.81 (0.20) | 0.84 (0.19) |
| eGFR (ml/min/1.73 m^2^) | 105.4 (27.7) | 103.5 (35.7) | 100.5 (33.1) | 99.9 (53.3) | 94.5 (42.9) |
| UAE (mg/24 hours)* | 8.09 (7.2) | 8.76 (8.37) | 9.61 (10.41) | 9.47 (12.06) | 10.96 (16.00) |
| GGT (U/L)* | 17 (12) | 21 (16) | 24 (22) | 26 (22) | 30 (30) |
| ALT (U/L)* | 16 (8) | 19 (11) | 21 (12) | 22 (13) | 22 (15) |

*, are median (interquartile range); ALT, alanine aminotransferase; ALP, alkaline phosphatase; Apo AI, apolipoprotein AI; Apo B, apolipoprotein; BMI, body mass index; hsCRP, high sensitivity C-reactive protein; DBP, diastolic blood pressure; eGFR, estimated glomerular filtration rate (as calculated using the Chronic Kidney Disease Epidemiology Collaboration combined creatinine-cystatin C equation); GGT, gamma-glutamyltransferse; HDL-C, high-density lipoprotein cholesterol; SBP, systolic blood pressure; UAE, urinary albumin excretion
