# Supplementary material for: Serum Alkaline Phosphatase and Risk of Incident Cardiovascular Disease: Interrelationship with High Sensitivity C-Reactive Protein
Source: PLoS One. 2015 Jul 13;10(7):e0132822. doi: 10.1371/journal.pone.0132822 (PMC4500413; doi:10.1371/journal.pone.0132822)
Supplement: S2 Table — (DOCX) [file pone.0132822.s004.docx]

**S2 Table.** **Hazard Ratios for cardiovascular disease with first two years of follow-up, participants with history of diabetes, participants on regular antihypertensive medication, participants on regular lipid-lowering medication, and participants with UAE ≥ 30 mg/24 hours excluded**

| **Quintiles of ALP** | **Events / Total** | **Model 1** |  | **Model 2** |  | **Model 3** |  | **Model 4** |  |
| --- | --- | --- | --- | --- | --- | --- | --- | --- | --- |
|  |  | HR (95% CI) | *P-*value | HR (95% CI) | *P-*value | HR (95% CI) | *P-*value | HR (95% CI) | *P-*value |
| Excluding the first two years of follow-up | | | | | | | | | |
| Q1 – Q4 | 481 / 5,595 | ref |  | ref |  | ref |  | ref |  |
| Q5 | 246 / 1,368 | 1.50 (1.29 to 1.76) | < 0.001 | 1.34 (1.14 to 1.56) | < 0.001 | 1.33 (1.13 to 1.55) | < 0.001 | 1.24 (1.05 to 1.45) | 0.009 |
| Excluding participants with a history of diabetes | | | | | | | | | |
| Q1 – Q4 | 445 / 5,476 | ref |  | ref |  | ref |  | ref |  |
| Q5 | 229 / 1,286 | 1.56 (1.32 to 1.83) | < 0.001 | 1.40 (1.19 to 1.65) | < 0.001 | 1.39 (1.18 to 1.64) | < 0.001 | 1.29 (1.09 to 1.53) | 0.003 |
| Excluding participants on regular anti-hypertensive medication | | | | | | | | | |
| Q1 – Q4 | 364 / 5,043 | ref |  | ref |  | ref |  | ref |  |
| Q5 | 183 / 1,170 | 1.52 (1.26 to 1.82) | < 0.001 | 1.31 (1.09 to 1.57) | 0.004 | 1.29 (1.07 to 1.55) | 0.007 | 1.22 (1.01 to 1.47) | 0.040 |
| Excluding participants on regular lipid-lowering medication | | | | | | | | | |
| Q1 – Q4 | 453 / 5,477 | ref |  | ref |  | ref |  | ref |  |
| Q5 | 231 / 1,321 | 1.47 (1.25 to 1.73) | < 0.001 | 1.31 (1.11 to 1.54) | 0.001 | 1.30 (1.10 to 1.53) | 0.002 | 1.21 (1.02 to 1.43) | 0.025 |
| Excluding participants with UAE ≥ 30 mg/24 hours | | | | | | | | | |
| Q1 – Q4 | 342 / 4,838 | ref |  | ref |  | ref |  | ref |  |
| Q5 | 178 / 1,188 | 1.47 (1.22 to 1.77) | < 0.001 | 1.34 (1.11 to 1.62) | 0.002 | 1.32 (1.10 to 1.59) | 0.004 | 1.25 (1.03 to 1.51) | 0.023 |

ALP, alkaline phosphatase; UAE, urinary albumin excretion; UAE, urinary albumin excretion; Q, quintile

Model 1: Age and sex
Model 2: Model 1 plus smoking status, history of diabetes, systolic blood pressure, total cholesterol, and high-density lipoprotein-cholesterol
Model 3: Model 2 plus body mass index, alcohol consumption, glucose, log_e_ triglycerides, estimated glomerular filtration rate (as calculated using the Chronic Kidney Disease Epidemiology Collaboration combined creatinine-cystatin C equation), and log_e_ urinary albumin excretion

Model 4: Model 3 plus log_e_ C-reactive protein
