# Supplementary material for: Serum Alkaline Phosphatase and Risk of Incident Cardiovascular Disease: Interrelationship with High Sensitivity C-Reactive Protein
Source: PLoS One. 2015 Jul 13;10(7):e0132822. doi: 10.1371/journal.pone.0132822 (PMC4500413; doi:10.1371/journal.pone.0132822)
Supplement: S3 Table — (DOCX) [file pone.0132822.s005.docx]

**S3 Table.** **Age and sex-adjusted hazard ratios of ALP for incident cardiovascular diseases with additional adjustment for C-reactive protein**

| **Quintiles of ALP** | **Events / Total** | **Model 1** |  | **Model 2** |  |
| --- | --- | --- | --- | --- | --- |
|  |  | HR (95% CI) | *P-*value | HR (95% CI) | *P-*value |
| Q1 – Q4 | 486 / 5,601 | ref |  | ref |  |
| Q5 | 251 / 1,373 | 1.51 (1.29 to 1.76) | < 0.001 | 1.32 (1.13 to 1.55) | 0.001 |

ALP, alkaline phosphatase; Q, quintile

Model 1: Age and sex

Model 2: Model 1 plus log_e_ C-reactive protein
