# Supplementary material for: Serum Alkaline Phosphatase and Risk of Incident Cardiovascular Disease: Interrelationship with High Sensitivity C-Reactive Protein
Source: PLoS One. 2015 Jul 13;10(7):e0132822. doi: 10.1371/journal.pone.0132822 (PMC4500413; doi:10.1371/journal.pone.0132822)
Supplement: S4 Table — (DOCX) [file pone.0132822.s006.docx]

**S4 Table.** **Association of alkaline phosphatase with incident cardiovascular disease, coronary heart disease, and stroke with further adjustment for other liver enzymes**

| **Quintiles of ALP** | **Events / Total** | **Model 1** |  | **Model 2** |  | **Model 3** |  | **Model 4** |  |
| --- | --- | --- | --- | --- | --- | --- | --- | --- | --- |
|  |  | **HR (95% CI)** | ***P-*value** | **HR (95% CI)** | ***P-*value** | **HR (95% CI)** | ***P-*value** | **HR (95% CI)** | ***P-*value** |
| Cardiovascular disease | | | | | | | | | |
| Q1 – Q4 | 486 / 5,599 | ref |  | ref |  | ref |  | ref |  |
| Q5 | 250 / 1,370 | 1.50 (1.29 to 1.75) | < 0.001 | 1.34 (1.14 to 1.56) | < 0.001 | 1.32 (1.13 to 1.55) | < 0.001 | 1.30 (1.11 to 1.52) | 0.001 |
| Coronary heart disease | | | | | | | | | |
| Q1 – Q4 | 172 / 4,187 | ref |  | ref |  | ref |  | ref |  |
| Q5 | 136 / 1,373 | 1.57 (1.25 to 1.98) | < 0.001 | 1.29 (1.03 to 1.63) | 0.029 | 1.30 (1.03 to 1.64) | 0.026 | 1.21 (0.95 to 1.53) | 0.122 |
| Stroke | | | | | | | | | |
| Q1 – Q4 | 83 / 4,187 | ref |  | ref |  | ref |  | ref |  |
| Q5 | 48 / 1,373 | 1.16 (0.76 to 1.79) | 0.494 | 1.04 (0.68 to1.61) | 0.848 | 1.05 (0.68 to 1.63) | 0.811 | 0.91 (0.59 to 1.42) | 0.684 |

ALP,alkaline phosphatase; Q, quintile

Model 1: Age and sex
Model 2: Model 1 plus smoking status, history of diabetes, systolic blood pressure, total cholesterol, and high-density lipoprotein-cholesterol
Model 3: Model 2 plus body mass index, alcohol consumption, glucose, log_e_ triglycerides, estimated glomerular filtration rate (as calculated using the Chronic Kidney Disease Epidemiology Collaboration combined creatinine-cystatin C equation), and log_e_ urinary albumin excretion

Model 4: Model 3 plus log_e_ gamma-glutamyltransferase and log_e_ alanine aminotransferase
