# Supplementary material for: Serum Alkaline Phosphatase and Risk of Incident Cardiovascular Disease: Interrelationship with High Sensitivity C-Reactive Protein
Source: PLoS One. 2015 Jul 13;10(7):e0132822. doi: 10.1371/journal.pone.0132822 (PMC4500413; doi:10.1371/journal.pone.0132822)
Supplement: S5 Table — (DOCX) [file pone.0132822.s007.docx]

**S5 Table.** **Risk discrimination and reclassification upon addition of ALP to a CVD risk prediction model containing Reynolds Risk Score components**

| **Discrimination** |  |
| --- | --- |
| C-index (95% CI): conventional risk factors | 0.7882 (0.7726 to 0.8039) |
| C-index (95% CI): conventional risk factors plus ALP | 0.7883 (0.7726 to 0.8039) |
| C-index change (95% CI) | 0.0001 (-0.0010 to 0.0011) |
| *P*-value | 0.96 |
|  |  |
| **Reclassification** |  |
| *Participants who did not develop CVD at 10 years* |  |
| Appropriately reclassified | 41 (0.90%) |
| Inappropriately reclassified | 57 (1.26%) |
| No change | 4,438 (97.84%) |
| *Participants who developed CVD at 10 years* |  |
| Appropriately reclassified | 7 (1.12%) |
| Inappropriately reclassified | 7 (1.12%) |
| No change | 613 (97.77%) |
|  |  |
| Net reclassification index (95% CI) | -0.35% (-1.60% to 0.89%) |
| *P*-value | 0.58 |
|  |  |

The Reynold Risk Score model included age, sex, smoking status, systolic blood pressure, total cholesterol, high-density lipoprotein cholesterol, high sensitivity C-reactive protein, and parental history of premature myocardial infarction; ALP, alkaline phosphatase; CVD, cardiovascular disease
